# Conversion of Oleic Acid into Azelaic and Pelargonic Acid by a Chemo-Enzymatic Route

**DOI:** 10.3390/molecules25081882

**Published:** 2020-04-18

**Authors:** Elisabetta Brenna, Danilo Colombo, Giuseppe Di Lecce, Francesco G. Gatti, Maria Chiara Ghezzi, Francesca Tentori, Davide Tessaro, Mariacristina Viola

**Affiliations:** 1 Dipartimento di Chimica, Materiali ed Ingegneria Chimica “Giulio Natta”, Politecnico di Milano, Via Mancinelli 7, 20131 Milano, Italy; danilo.colombo@polimi.it (D.C.); francesco.gatti@polimi.it (F.G.G.); mariachiara.ghezzi@polimi.it (M.C.G.); francesca.tentori@polimi.it (F.T.); davide.tessaro@polimi.it (D.T.); mariacristina.viola@polimi.it (M.V.); 2 Oleificio Zucchi S.p.A., Via Acquaviva, 26100 Cremona, Italy; dirlab@oleificiozucchi.com

**Keywords:** lipase, biocatalysis, unsaturated fatty acid, oxidative cleavage, oxidation

## Abstract

A chemo-enzymatic approach for the conversion of oleic acid into azelaic and pelargonic acid is herein described. It represents a sustainable alternative to ozonolysis, currently employed at the industrial scale to perform the reaction. Azelaic acid is produced in high chemical purity in 44% isolation yield after three steps, avoiding column chromatography purifications. In the first step, the lipase-mediated generation of peroleic acid in the presence of 35% H_2_O_2_ is employed for the self-epoxidation of the unsaturated acid to the corresponding oxirane derivative. This intermediate is submitted to in situ acid-catalyzed opening, to afford 9,10-dihydroxystearic acid, which readily crystallizes from the reaction medium. The chemical oxidation of the diol derivative, using atmospheric oxygen as a stoichiometric oxidant with catalytic quantities of Fe(NO_3_)_3_∙9∙H_2_O, (2,2,6,6-tetramethylpiperidin-1-yl)oxyl (TEMPO), and NaCl, affords 9,10-dioxostearic acid which is cleaved by the action of 35% H_2_O_2_ in mild conditions, without requiring any catalyst, to give pelargonic and azelaic acid.

## 1. Introduction

The employment of renewable feedstocks in the chemical industry is steadily advancing to ensure more efficient use of natural resources, reduce the dependence on fossil raw materials, and give a contribution to achieving sustainable consumption and production patterns [1]. 

**Figure 1:**
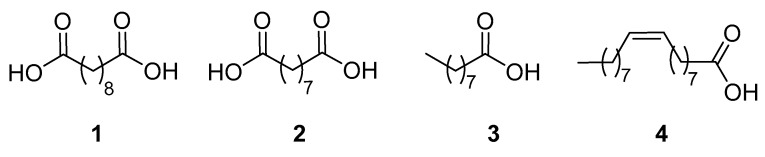
Sebacic acid (**1**), azelaic acid (**2**), pelargonic acid (**3**), and oleic acid (**4**).

Fats and oils represent an important class of renewable feedstock from which the so-called oleochemicals are obtained. They are abundant in nature, biodegradable, and have nontoxic properties. They have long hydrocarbon chains, resembling the structure of petroleum components, but they are also characterized by several functional groups useful for chemical modification. The major process for transforming fats and oils into oleochemicals is hydrolysis, converting natural triglycerides into crude glycerine and mixtures of fatty acids. The latter are then submitted to reactions involving either the carboxylic group (to afford soaps, esters, amides, amines, and alcohols) or the reduction/oxidation of the C=C double bonds, if present. Among these procedures developed to obtain fine chemicals, the oxidative cleavage of unsaturated fatty acids for the production of dicarboxylic acids, hydroxy acids, and amino acids has received great attention in the last decade [2,3,4]. Until recently, only two dicarboxylic acids prepared from oleochemicals have been commercialized, i.e., sebacic acid (**1**), obtained by the alkaline cleavage of castor oil [5], and azelaic acid (**2**), which is produced together with pelargonic acid (**3**) by ozonolysis of oleic acid (**4**) (Figure 1) [6]. Sebacic and azelaic acid are extensively employed in the synthesis of new generation biodegradable copolymers [7]. Azelaic acid, naturally occurring in wheat, rye, and barley, also finds application as an active ingredient in products for the topical treatment of acne [8], and for the stimulation of hair regrowth [9]. It works by inhibiting the growth of skin bacteria causing acne, and by keeping skin pores clear. Pelargonic acid, found in nature as ester derivatives in the oil of pelargonium, is used as an herbicide to prevent the growth of weeds both indoors and outdoors, and as a blossom thinner for apple and pear trees [10].

Oleic acid is the most abundant monounsaturated fatty acid in nature [11], present in a wide range of vegetable and animal oils and fats. Several works have been published in recent years describing alternative methods to the ozone-promoted oxidative scission, most of which are based on metal catalysis [12]. Among them, some very effective one-pot procedures involve the use of H_2_O_2_ as primary oxidant in the presence of tungsten derivatives: (i) methyltrioctylammonium tetrakis(oxodiperoxotungsto)phosphate [13] (40% H_2_O_2_, 85 °C, yields of compounds **2** and **3** by GC/MS analysis of the crude mixture were 79% and 82%, respectively); (ii) WO_3_ and Na_2_SnO_3_ in *t*-BuOH [14] (31% H_2_O_2_, 130 °C, sealed glass vial, isolation yields were 89% and 65% for **2** and **3**, respectively); (iii) a new hybrid organic/inorganic polyoxotungstate in *t*-BuOH [15] (30% H_2_O_2_, 120 °C, yield of compounds **2** and **3** by GC/MS analysis of the crude mixture were 79% and 80%, respectively); iv) H_2_WO_4_ [16] (60% H_2_O_2_, reflux, isolation yield was 60% for **2**); (v) Na_2_WO_4_ aqueous solution/H_3_PO_4_ aqueous solution with a suitable phase transfer catalyst in a sealed flask [17] (30% H_2_O_2_, 90 °C, yield of compound **2** by GC/MS analysis of the crude mixture was max 54%); (vi) the peroxo–tungsten complex [C_5_H_5_N(*n*-C_16_H_33_)]_3_{PO_4_[WO(O_2_)_2_]_4_} as a phase-transfer catalyst/co-oxidant [18] (30% H_2_O_2_, 85 °C, yields of compounds **2** and **3** by GC/MS analysis of the crude mixture were 79% and 80%, respectively), using in this case oleic acid obtained upon hydrolysis of high oleic sunflower oil by *Candida cylindracea* lipase.

**Figure 2:**
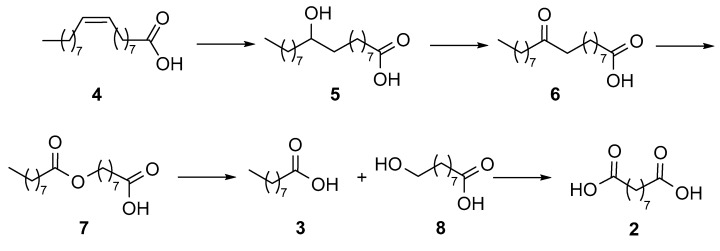
Synthesis of azelaic acid (**2**) from oleic acid (**4**) according to references [19,20].

As for biocatalytic methods, only a few reports have appeared in the literature. Song et al. developed [19,20] a multi-step enzymatic procedure (Figure 2) starting from the hydration of oleic acid (**4**), followed by the oxidation of the intermediate alcohol **5** to the ketone derivative **6** that was in turn submitted to Baeyer–Villiger (BV) oxidation to afford ester **7**. The latter was hydrolyzed to afford acid **3** and the hydroxy acid **8**, which was finally oxidized to azelaic acid (**2**). 

In [19] the possibility to obtain a regioisomer of ester **7** giving directly diacid **2** upon hydrolysis was described, but it was not considered for further study and optimization in the following publication [20]. The same research group described the preparation of azelaic acid from ricinoleic acid [21]. The use of linoleic acid for the biocatalyzed production of azelaic acid was reported by Hauer et al. [22,23]. A multi-enzymatic one-pot reaction was developed to convert linoleic acid into azelaic acid by combining a 9*S*-lipoxygenase and 9/13-hydroperoxide lyase to obtain 9-oxononanoic acid submitted to the final oxidation to acid **2** catalyzed by an alcohol dehydrogenase. In 2019 the capability of *Candida tropicalis* ATCC20962 to transform nonanoic acid and its esters into azelaic acid **2** with the aid of nonane addition and continuous glucose supply [24] was investigated, to improve the production yield of diacid **2** obtained in the ozonolysis process of oleic acid. 

Recently, we were involved in a project aimed at the valorization of the side-stream products generated by an Italian plant for vegetable seed oil refining (Oleificio Zucchi, Cremona) by using biocatalytic methods. An important step of the refining process (neutralization) is represented by the removal of free fatty acids, producing a side-product, called soapstock, which is currently disposed of by Zucchi in bio-digesters. The fatty acids profile of this material depends on the nature of the vegetable oil, and, in particular, the one obtained from sunflower oil is highly enriched in oleic acid [25]. Thus, we started to investigate a novel chemo-enzymatic oxidative scission of oleic acid, to be applied to the sunflower soapstock coming from Oleificio Zucchi for its valorization. The preliminary results of this study were obtained while working on commercial oleic acid, as a model compound, to study each step of the most suitable synthetic procedure more easily using a less complex starting material. The results are herein reported. 

## 2. Results

The enzymatic synthesis of azelaic acid reported in 2013 by Song et al. [19] (Figure 2) consisted of the use of recombinant *Escherichia coli* cells expressing at the same time the genes encoding an oleate hydratase from *Stenotrophomonas maltophilia*, an alcohol dehydrogenase (ADH) from *Micrococcus luteus*, and a BV monooxygenase (BVMO) from *Pseudomonas putida* KT2440 for the transformation of oleic acid into 9-(nonanoyloxy)nonanoic acid (**7**). The hydrolysis of this latter compound, mediated by a cell extract of *E. coli* expressing the esterase gene from *P. fluorescens*, afforded pelargonic acid (**3**) and 9-hydroxynonanoic acid (**8**). In a further development of the work [20], the oxidation of derivative **8** by an ADH from *P. putida* GPo1 completed the route to azelaic acid. As the final product concentration in the reaction medium was only a few millimolar, likely because of the toxic effects of pelargonic acid on the *E. coli* cells, the authors investigated the hydrolysis of **7** and the subsequent oxidation of derivative **8** into acid **2** by chemical methods [26]. The ester intermediate **7** was purified by extraction and column chromatography, hydrolyzed with sodium hydroxide in methanol/water (4/1) at 60 °C to afford 9-hydroxynonanoic acid **8**, which was separated from pelargonic acid by column chromatography. Finally, the oxidation of the terminal hydroxy group of derivative **8** was achieved using NaClO_2_ (1.2 equiv.), 2,2,6,6-tetramethyl-piperidin-1-yl oxyl (TEMPO) (4 mol%), and NaOCl (2 mol%) in aqueous acetonitrile. After these two steps, no purification was needed. The overall molar yield of azelaic acid from oleic acid was 58%.

**Figure 3:**
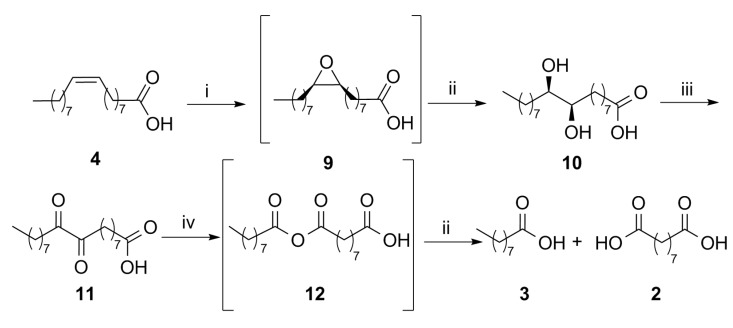
The synthesis of azelaic acid (**2**) from oleic acid (**4**) described in this paper. (i) H_2_O_2_ 35%, Novozyme 435, acetonitrile, 5 h, 50 °C; (ii) NaHSO_3_ saturated solution, H_2_SO_4_ 2 M, 3 h, r.t.; (iii) atmospheric O_2_, cat. Fe(NO_3_)_3_∙9 H_2_O/TEMPO/NaCl, toluene, 5 h, 100 °C; (iv) 35% H_2_O_2_, toluene, 3 h, 30 °C.

We adopted a different strategy (Figure 3), consisting of the epoxidation of oleic acid to derivative **9**, followed by the formation of *threo*-9,10-dihydroxystearic acid (**10**) due to the acid-catalyzed hydrolytic cleavage of the oxirane ring, promoted directly in the epoxidation medium. The chemical oxidation of the diol afforded 9,10-dioxostearic acid (**11**), which was submitted to oxidative cleavage to afford a mixture of pelargonic (**3**) and azelaic (**2**) acid, through the intermediate anhydride **12**. In a recent publication [27], the oxidation of the methyl ester derivative of compound **10**, using a solvent-free procedure of dehydrogenative oxidation catalyzed by commercial 64 wt.% Ni/SiO_2_ in the presence of 1-decene as a scavenger_,_ was employed to afford a mixture of the two possible regioisomeric vicinal ketols, that were successively cleaved with formic acid/hydrogen peroxide, and afforded up to 80% pelargonic acid and azelaic acid monomethyl ester.

### 2.1. Epoxidation of Oleic Acid *(**4**)* to 8-(3-Octyloxiran-2-yl)Octanoic Acid *(**9**)*

The capability of certain lipases to catalyze the perhydrolysis (i.e., lysis by hydrogen peroxide) of carboxylic acid esters, hence forming peroxycarboxylic acids in aqueous hydrogen peroxide solutions, had been already patented by Clorox co. in the late eighties [28]. In 1990, an immobilized form of lipase B from *Candida antarctica* (Novozyme 435) was shown [29] to catalyze the formation of peroxycarboxylic acids directly from the corresponding carboxylic acid. In that case, the reaction was combined with the epoxidation of alkenes. A few years later, Warwel et al. [30] described that, when unsaturated fatty acids (or their esters) are treated with hydrogen peroxide in the presence of Novozyme 435, epoxidized derivatives are obtained through two sequential steps. Firstly, unsaturated fatty acids are converted into unsaturated peroxy acids by lipase-catalyzed perhydrolysis. Unsaturated peroxy or carboxylic acids are in turn epoxidized via a classical Prileshajev reaction, which is, in this case, referred to as a “self-epoxidation reaction” even though it proceeds predominantly via an intermolecular process. 

The reaction has been widely exploited not only for the epoxidation of fatty acids and esters but also for the derivatization of vegetable oils [31]. Typically, the reaction medium consists of an aqueous layer containing hydrogen peroxide, an organic layer (usually a toluene solution) containing the fatty acid derivative, and a solid phase represented by the immobilized enzyme. The main issue of this chemo-enzymatic procedure is the deactivation of lipase. Temperature, reaction time, concentration of H_2_O_2_ in the reaction medium, and the related concentration of peracid generated in situ are critical parameters to be considered. Temperatures not higher than 50 °C, diluted H_2_O_2_ solution (max 1% *w*/*w* in the final solution) and reaction times not longer than 6 h represent the most common experimental conditions. 

We decided to perform the chemo-enzymatic epoxidation of oleic acid in a water-miscible solvent, such as acetonitrile, for the following reasons: i) to promote the dissolution of both oleic acid and H_2_O_2_ in the same medium, and ii) to enable the in situ acid-catalyzed hydrolysis of the epoxide derivative at the end of the reaction, after removal of the enzyme, by addition of a diluted solution of sulfuric acid. The preliminary experiments were carried out with 0.30 mmol of commercial oleic acid (91% purity by GC/MS, the major contaminants are palmitic and stearic acid), changing the molar ratio H_2_O_2_/oleic acid (1.8 and 2.2), the temperature (30 °C and 50 °C), the amount of Novozyme 435 (10 mg and 30 mg), and the solvent volume (2 mL and 6 mL). The reactions were monitored by GC/MS. The results of this screening are reported in Tables S1 and S2 (see Supplementary Material). The following conditions were found to be optimal for running the reaction: 0.15 M oleic acid, 0.27 M H_2_O_2_, 5 mg∙mL^−1^ Novozyme 435, in acetonitrile at 50 °C for 5 h under stirring with final 98% conversion (GC/MS,). Epoxide **9** could be recovered from the reaction mixture at 83% isolation yield (see Supplementary Material), starting from commercial oleic acid. The stereochemistry of the oxirane ring of derivative **9** was based on the *cis* configuration of oleic acid and was confirmed by comparison with literature data (see Supplementary Material).

### 2.2. Acid–Catalyzed Cleavage of 8-(3-Octyloxiran-2-yl)Octanoic Acid *(**9**)* to 9,10-Didhydroxystearic Acid *(**10**)*

The ring-opening of epoxystearic acid **9** to the corresponding *threo* diol derivative **10** (Figure 3) was promoted by diluted sulfuric acid, and the reaction conditions were adjusted to obtain quantitative conversion after 3 h at room temperature (Table S3 of Supplementary Material). The relative configuration of diol **10** was established following the *anti* mechanism of the hydrolytic opening of the oxirane ring of *cis*-epoxide **9** and was confirmed by literature data (see Materials and Methods). The acid-catalyzed hydrolysis was then performed, without isolation of the epoxide, by addition of a 2.0 M solution of H_2_SO_4_ directly to the reaction mixture of the epoxidation step, after removal of the enzyme by filtration and decomposition of peroxy species. This one-pot two-steps sequence led to the spontaneous crystallization of dihydroxystearic acid **10**, which was easily recovered from the reaction medium by filtration as a pure white solid. Satisfactory isolation yields (77%) were obtained, even when the reaction was run at a 2 g scale, starting from commercial 91% oleic acid.

### 2.3. Oxidation of 9,10-Dihydroxystearic Acid *(**10**)* to 9,10-Dioxostearic Acid *(**11**)*

The following procedures were tested to achieve the oxidation of either one or both the hydroxy groups of derivative **10**: (i) alcohol dehydrogenase—mediated oxidation (commercial kit from EVOXX); (ii) chemo-enzymatic oxidation with laccase and hydroxybenzotriazol (HOBt); (iii) aerobic oxidation with catalytic Fe(NO_3_)_3_∙9 H_2_O, TEMPO, and NaCl. Only the latter was successful and diol **10** could be converted into the corresponding dioxo derivative **11** (Figure 3). Starting from 0.50 g of diol **10**, according to the literature [32], a loading of 1 mol% for each catalyst was enough to afford complete conversion into the diketone in toluene solution at 100 °C in 5 h. After work-up (quenching with water and extraction), the crude residue was submitted directly to the following step of oxidative cleavage.

### 2.4. Oxidative Cleavage of 9,10-Dioxostearic Acid *(**11**)* to Azelaic *(**2**)* and Pelargonic Acid *(**3**)*

For the final step of the synthetic procedure, we investigated the Baeyer–Villiger (BV) oxidation of diketone **11**, to prepare the corresponding anhydride **12**, and hydrolyze it to azelaic (**2**) and pelargonic acid (**3**). A wide range of oxidants has been employed for the BV reaction, including mineral and organic peracids. Hydrogen peroxide can be used if suitably activated by a catalyst, or in the presence of a strong acid, or even in alkaline conditions [33,34]. α-Diketones react readily with BV reagents: in inert solvents anhydrides are formed, while in alkaline or acidic media simple carboxylic acids are generally produced in good yields [35]. In 1930 Böeseken et al. [36] prepared 9,10-diketostearic acid (**11**) by oxidation of 9-octadecynoic acid with 70% nitric acid at 10–25% yield and submitted it to the reaction with 15% excess peracetic acid in acetic acid for one day. They obtained the quantitative conversion of the diketone into a mixture of acids **2** and **3.**

Thus, we first considered the possibility to perform the BV oxidation of compound **11** with the corresponding peroxycarboxylic acid produced by lipase-mediated perhydrolysis in the presence of H_2_O_2_, using for preliminary experiments a sample of **11** isolated and purified by column chromatography. We treated dioxostearic acid **11** (50 mg) with 1.6 mol of H_2_O_2_ per mol of dioxostearic acid in the presence of 2.5 mg∙mL^−1^ Novozyme 435 in toluene (2 mL), the same solvent employed for the preceding oxidation. The cleavage was complete after 3 h at 30 °C, to give a mixture of 96% pelargonic and azelaic acids, with tiny quantities of octanoic (0.3%), stearic (0.4%), and palmitic acid (2%), with other minor components (GC/MS). To our surprise, when the blank reaction was carried out in parallel in identical conditions without the presence of the enzyme, the same oxidative cleavage was observed, affording a mixture containing acids **2** and **3**, besides 51% of intermediate anhydride **12** (^1^H NMR). The presence of intermediate **12** was highlighted by NMR spectroscopy. The ^13^C NMR spectrum of the crude reaction mixture showed the presence of two singlets at 169.73 and 169.67 ppm for the carboxylic carbon atoms of the anhydride, next to those around 180 ppm which belong to acids **2** and **3** and to the COOH group of compound **12.** In the ^1^H NMR spectrum, the triplet of the CH_2_ groups linked to the CO-O-CO moiety is at 2.44 ppm, a little more deshielded than the triplet of the CH_2_ beside COOH in compounds **2** and **3** and in the anhydride itself, occurring at 2.35 ppm. The ^1^H and ^13^C NMR spectra of anhydride **12** are not known in the literature, and the spectroscopic data of lauric anhydride reported in [37] were used as reference data. 

The reaction was repeated in acetonitrile and only 11% of anhydride was found in the final mixture. When the oxidation was carried out in toluene and 2 M H_2_SO_4_ was added to the reaction mixture during the workup procedure, after having decomposed peroxy species with NaHSO_3_ saturated solution, complete hydrolysis of intermediate **12** was obtained.

After investigation of every single step, the whole procedure was performed starting from 2 g of commercial oleic acid. Acidic hydrolysis was performed soon after epoxidation in a one-pot procedure, to afford diol derivative **10** as a pure compound at 70% isolated yield by filtration of the first crop of crystalline material and recovery of other product by further treatment of the mother liquors. The oxidation to dioxoderivative **11** gave a crude compound (75% purity by GC/MS) that was submitted directly to the last step of oxidative cleavage in toluene with only 35% H_2_O_2_, to provide a mixture of azelaic and pelargonic acids. Diacid **2** was recovered following a procedure, which had been already described in the literature [16], and based on the solubility of compound **2** in hot water. Repartition between ethyl acetate and hot water afforded an aqueous phase from which azelaic acid crystallised upon cooling. After three extraction cycles, diacid **2** could be recovered as a pure compound in 73% yield. Pelargonic acid **3** was isolated from the organic phase at 77% isolation yields, showing 91% chemical purity (GC/MS). The separation of diacid **2** from compound **3** was also investigated by using column chromatography, eluting with hexane–EtOAc mixtures with an increasing amount of the more polar solvent (see Supplementary Material), affording pure **2** and **3** in slightly higher isolation yields (81% and 84%, respectively).

## 3. Discussion

Ozonolysis of alkene bonds is a useful chemical transformation which is employed not only at the laboratory level but also at industrial scale for the rapid and effective oxidative cleavage of C=C double bonds [38]. The primary concern related to ozonolysis chemistry is represented by the serious safety issues connected with the reaction, and in particular with the explosive hazard due to the instability of intermediate ozonides. The present industrial production of azelaic acid is entirely based on ozonolysis of oleic acid, being the global azelaic acid market valued at 94 million USD in 2017 and expected to reach 140 million USD by 2025 [39]. The market is mainly driven by growing demand for plastics and lubricants, which hold above 70% of global azelaic acid consumption. 

The growing attention towards the development of safer and more environmentally friendly production technologies has stimulated the investigation of alternative methods for the conversion of oleic acid into azelaic acid. Suitable references have been reported in the Introduction. We gave a contribution to this search by investigating a chemo-enzymatic approach to achieve the target oxidative scission. 

We decided to use the in-situ peroxidation of oleic acid **4** by lipase-mediated perhydrolysis in the presence of hydrogen peroxide 35% as a safe procedure to afford the peroxycarboxylic acid needed to promote the epoxidation step at the beginning of the synthetic sequence. Oleic acid itself undergoes the conversion into the reactive peroxy species, so it is possible to avoid the use of an additional carboxylic acid that would remain in the reaction mixture as a by-product to be removed from the desired final compound. The advantage of the proposed procedure is also that storage and manipulation of peracid are avoided: it is generated in the reaction medium, and the excess is destroyed at the end of its use. The best biocatalyst for this reaction is Novozyme 435 which has the advantage of being an immobilized form of *C. Antarctica* B that can be recovered and re-used. Preliminary experiments were performed starting from 1 g of oleic acid, and recovering the enzyme by filtration and washing with water and acetonitrile. The enzyme was kept at 4 °C for 18 h and re-used in a subsequent reaction. After four runs the conversion of oleic acid (GC/MS analysis) into the epoxide was 78%. The evaluation of the enzyme reusability in these experiments can only be used as a first orientation because it is influenced by the effects of manipulation and storage on the enzyme support, overlapping those due to hydrogen peroxide and peracid. Long-term performance of the enzyme should be best studied under continuous process conditions, as suggested by recent literature [40]. Very positive results on the stability of this lipase in this type of reaction have been obtained using both a packed-bed reactor [41] and a continuous stirred tank reactor [40]. This kind of investigation is now in progress in our research group.

We considered also the possibility to avoid the isolation and purification of some of the intermediates of our procedure to reduce quantities of waste, solvents and separation aids. We chose the solvent of the epoxidation reaction to telescope the first two steps of the procedure, and perform the acid-catalyzed opening of the oxirane ring without isolation of epoxide **9**. Diol derivative **10** could be obtained as a pure crystalline compound by crystallization from acetonitrile, after quenching the peroxy species with NaHSO_3_ and promoting epoxide cleavage with catalytic H_2_SO_4_ aqueous solution. No column chromatography was needed, thus favoring the isolation yield of diol **10** and limiting further use of solvents. Even the purification of dioxostearic acid **11** could be avoided, and the raw material was submitted directly to the following step to afford azelaic and pelargonic acid. The use of toluene as a solvent for both the diol oxidation and the final oxidative cleavage reaction will be useful in the future for developing the procedure in continuous flow mode. 

For the last step of the whole sequence, we discovered the unexpected capability of H_2_O_2_ 35% w/w to promote the oxidative cleavage of diketone **11** in organic solvents, either toluene or acetonitrile, at 30 °C, without the addition of any catalyst. H_2_O_2_ is considered as a green oxidant, generating water as a by-product. It is safely stored and transported, and easily available on the market at a cheap price.

## 4. Materials and Methods 

### 4.1. General Methods

Chemicals and solvents were purchased from Merck (Merck Life Science S.r.l., Milan, Italy) and used without further purification. Trimethylsilyldiazomethane 10% solution in hexane (TCI Europe N.V.) was purchased from Zentek Srl (Milan, Italy). Novozyme 435 (Novozymes) was purchased from Strem Chemicals Inc. (Bischheim, France). TLC analyses were performed on Macherey Nagel pre-coated TLS sheets Polygram^®^ SIL G/UV_254_ purchased from Chimikart s.r.l. (Naples, Italy). ^1^H and ^13^C NMR spectra were recorded on a 400 or 500 MHz spectrometer in CDCl_3_ solution at r.t. The chemical shift scale was based on internal tetramethylsilane. GC/MS analyses were performed using an HP-5MS column (30 m × 0.25 mm × 0.25 μm, Agilent Technologies Italia Spa, Cernusco sul Naviglio, Italy). The following temperature program was employed: 50 °C/10 °C min^−1^/250 °C (5 min)/50 °C min^−1^/300 °C (10 min). The samples for GC/MS were treated with MeOH and trimethylsilyldiazomethane 10% in hexane, to derivatize carboxylic acids by transformation into the respective methyl esters.

### 4.2. One-Pot Two-Step Synthesis of Threo-9,10-Didhydroxystearic Acid *(**10**)*

A suspension of Novozyme 435 (240 mg) in acetonitrile (48 mL), containing oleic acid (2.2 g, 91% purity, 7.1 mmol) and 35% H_2_O_2_ w/w (1.1 mL, 12.8 mmol) was shaken in an orbital shaker (160 rpm, 50 °C) for 5 h. The enzyme was removed by filtration, washed with acetonitrile, and stored at 4 °C to be re-used. A saturated solution of NaHSO_3_ (2 mL) was added to the filtrate, followed by the addition of 2 M H_2_SO_4_ (963 μL). After 15 h at room temperature, diol **10** was recovered by filtration (1.62 g, 72%, sum of two crystallization crops). ^1^H NMR (CD_3_OD, 400 MHz) [42]: δ = 3.45–3.35 (2H, m, 2CHOH), 2.29 (2H, t with *J* = 7.4 Hz, CH_2_COOH), 1.70–1.15 (26H, m, 13 CH_2_), 0.97–0.82 (3H, m, CH_3_). ^13^C NMR (CD_3_OD, 100.6 MHz) [42]: δ = 177.6, 75.29, 75.26, 35.0, 34.0, 33.9, 33.0, 30.8, 30.7, 30.6, 30.42, 30.37, 30.2, 27.04, 26.96, 26.1, 23.7, 14.4. GC/MS (EI) as a methyl ester, obtained by treatment with MeOH and trimethylsilyldiazomethane 10% in hexane, t_r_ = 23.85 min: m/z (%) = 294 (M^+^ – 36, 1), 187 (48), 155 (100), 138 (30). 

### 4.3. Oxidation of 9,10-Dihydroxystearic Acid *(**9**)* to 9,10-Dioxostearic Acid *(**10**)*

A mixture of diol **10** (1.55 g, 4.9 mmol), Fe(NO_3_)_3_∙9 H_2_O (20 mg, 0.049 mmol), TEMPO (8.0 mg, 0.049 mmol), and NaCl (3 mg, 0.049 mmol) in toluene (45 mL) was stirred at 100 °C for 4–5 h. The reaction mixture was poured into water and extracted with ethyl acetate. The organic phase was dried and concentrated under reduced pressure to give the crude dioxo derivative **11** (1.83 g, 75% purity by GC/MS analysis, estimated content of compound **11** 1.37 g) which was employed in the successive step without further purification. ^1^H NMR (CDCl_3_, 400 MHz) [43]: δ = 2.72 (4H, t with *J* = 7.3 Hz, 2CH_2_CO), 2.35 (2H, t with *J* = 7.4 Hz, CH_2_COOH), 1.69–1.49 (6H, m, 3 CH_2_), 1.40–1.20 (16H, m, 8 CH_2_), 0.92–0.84 (3H, m, CH_3_). ^13^C NMR (CDCl_3_, 100.6 MHz) [43]: δ 200.3, 200.2, 180.0, 36.2, 36.1, 34.2, 31.9, 29.4, 29.3, 29.2, 29.1, 29.04, 28.96, 24.2, 23.2, 23.1, 22.8, 14.2. GC/MS (EI) as a methyl ester, obtained by treatment with MeOH and trimethylsilyldiazomethane 10% in hexane, t_r_ = 22.48 min: *m/z* (%) = 326 (M^+^, 1), 295 (5), 185 (100), 141 (54). 

### 4.4. Oxidative Cleavage of 9,10-Dioxostearic Acid *(**11**)* to Azelaic (***2***) and Pelargonic Acid *(**3**)*

A mixture of crude dioxo derivative **11** (1.75 g, 75% purity, estimated content of compound **11** 4.21 mmol) and 35% H_2_O_2_ w/w (579 μL, 6.73 mmol) in toluene (35 mL) was stirred at 30 °C for 3 h. A saturated solution of NaHSO_3_ (750 μL) was added, followed by the addition of H_2_SO_4_ 2 M till pH = 2. The reaction mixture was then extracted with ethyl acetate. The organic phase was dried and concentrated under reduced pressure to give a crude mixture containing 93% (GC/MS) of acids **2** and **3**, which was heated at 50 °C for 1 h in a 1:1 mixture of EtOAc and water. Water was separated, and diacid **2** crystallized upon cooling. Other two extractions of the organic phase with hot water allowed the isolation of diacid **2** as a pure compound. Pelargonic acid **3** was isolated from the organic phase showing 91% chemical purity (GC/MS). 

Azelaic acid (**2**): 578 mg (73%, >99% chemical purity by GC/MS and NMR); ^1^H NMR (CDCl_3_, 400 MHz) [44]: δ = 2.35 (4H, t with *J* = 7.4 Hz, 2CH_2_COOH), 1.75–1.55 (4H, m, 2CH_2_), 1.4–1.2 (6H, m, 3CH_2_). ^13^C NMR (CDCl_3_, 100.6 MHz) [44]: δ = 180.2, 34.2, 29.0, 28.9, 24.3. GC/MS (EI) as a methyl ester, obtained by treatment with MeOH and trimethylsilyldiazomethane 10% in hexane, t_r_ = 13.9 min: *m*/*z* (%) = 185 (M^+^ - 31, 55), 152 (100), 143 (47), 111 (63). 

Pelargonic acid (**3**): 512 mg (77%, 91% chemical purity by GC/MS); ^1^H NMR (CDCl_3_, 400 MHz) [45]: δ = 2.35 (2H, t with *J* = 7.5Hz, CH_2_COOH), 1.75–1.55 (2H, m, CH_2_), 1.4–1.2 (10H, m, 5CH_2_), 0.80–0.95 (3H, m, CH_3_). ^13^C NMR (CDCl_3_, 100.6 MHz) [45]: δ = 180.5, 34.2, 31.9, 29.3, 29.20, 29.18, 24.8, 22.7, 14.2. GC/MS (EI) as a methyl ester, obtained by treatment with MeOH and trimethylsilyldiazomethane 10% in hexane, t_r_ = 9.33 min: *m*/*z* (%) = 172 (M^+^, 0.5), 141 (15), 129 (18), 87 (45), 74 (100).

## 5. Conclusions

The chemo-enzymatic conversion of oleic acid into azelaic and pelargonic acids herein described represents a sustainable alternative to ozonolysis, currently employed at the industrial scale. Azelaic acid can be produced in high chemical purity in 44% isolation yield after three steps, avoiding column chromatography purifications. Intermediate diol **10** and final azelaic acid **2** are purified by crystallization from acetonitrile and water, respectively. The procedure shows some valuable aspects, even if it is not a one-pot process, as those using H_2_O_2_ and tungsten derivatives already known in the literature [13,14,15,16,17,18].

The reagents of the three steps are: (i) H_2_O_2_ 35% for the epoxidation of acid **4** and the oxidative cleavage of diketone **11**, and atmospheric oxygen for diol **10** oxidation, both producing H_2_O as a by-product; (ii) H_2_O for the acid-catalyzed hydrolysis of both epoxide **9** and anhydride **12,** generating no side-product, being fully incorporated in the reacting products. The organic solvents used during the reactions are limited to acetonitrile and toluene; water and ethyl acetate are employed for quenching and separation procedures. The final oxidative cleavage of dioxo derivative **11** occurs in mild conditions and generates a very tiny quantity of oxidized impurities, thus increasing the economic value of the process, and reducing the complexity and cost of final azelaic acid purification. Hydrogen peroxide is itself very effective in promoting the cleavage with no need for catalysts or harsh acidic or alkaline conditions and generating water as a side-product. The reaction medium can be either toluene or acetonitrile. The use of an enzymatic method to produce in situ H_2_O_2_ will be considered for further development of the process. 

Studies are now in progress to apply this synthetic procedure to soapstock recovered from the neutralization step during vegetable seed oil refining at Oleificio Zucchi. A pre-treatment step has to be added where the lipase-mediated hydrolysis of the triglycerides, which are inevitably present in this by-product, is carried out.

A further development of the process will also be the optimization of the entire sequence in continuous flow mode, taking advantage of the fact the enzyme employed for the generation of the key peroxy species is already marketed in immobilized form. Besides an expected higher productivity value and an advantage for the process scalability study, the use of a continuous flow reactor will most likely increase the stability of lipase, and allow for a more reliable evaluation of lipase reusability.

## Supplementary Materials

The following are available online, Table S1: Effect of H_2_O_2_/oleic acid molar ratio and temperature on the chemo-enzymatic epoxidation of oleic acid, Table S2: Effect of the amount of lipase and solvent on the chemo-enzymatic epoxidation of oleic acid, Table S3: Effect of the amount of aqueous 2M sulfuric acid on the ring-opening of epoxystearic acid **9**, Characterization of 8-((2*SR*,3*RS*)-3-octyloxiran-2-yl)octanoic acid (**9**), Recovery and re-use of Novozyme 435, Procedure for the oxidative cleavage of 9,10-dioxostearic acid (**11**) to give a mixture of 9-(nonanoyloxy)-9-oxononanoic acid (**12**), azelaic (**2**), and pelargonic acid (**3**), Column chromatography separation of azelaic (**2**) and pelargonic acid (**3**).
