# Supplementary material for: Conversion of Oleic Acid into Azelaic and Pelargonic Acid by a Chemo-Enzymatic Route
Source: Molecules. 2020 Apr 18;25(8):1882. doi: 10.3390/molecules25081882 (PMC7221618; doi:10.3390/molecules25081882)
Supplement: Supplementary file 1 [file molecules-25-01882-s001.pdf]

Supplementary Materials:

Conversion of oleic acid into azelaic and pelargonic acid by a chemo-enzymatic route

Elisabetta Brenna[1,]*,Danilo Colombo[1], Giuseppe Di Lecce[2], Francesco G. Gatti[1], Maria Chiara Ghezzi[1], Francesca Tentori[1], Davide Tessaro[1] and Mariacristina Viola[1]

[1] Dipartimento di Chimica, Materiali ed Ingegneria Chimica "Giulio Natta", Politecnico di Milano, Via Mancinelli 7, 20131, Milano, Italy; danilo.colombo@polimi.it (D.C.); francesco.gatti@polimi.it (F.G.G.); mariachiara.ghezzi@polimi.it (M.C.G.); francesca.tentori@polimi.it (F.T.); davide.tessaro@polimi.it (D.T.); mariacristina.viola@polimi.it (M.V.).

[2] Oleificio Zucchi S.p.A., Via Acquaviva, Cremona, Italy; dirlab@oleificiozucchi.com

* Correspondence: mariaelisabetta.brenna@polimi.it ; Tel.: +39-02-2399-3077

† The authors are listed in alphabetical order.

Epoxidation of oleic acid (4) to 8-(3-octyloxiran-2-yl)octanoic acid (9)

Table S1. Effect of H_2O_2/oleic acid molar ratio and temperature on the chemo-enzymatic epoxidation of oleic acid

oleic acid	lipase	reaction time	acetonitrile
93 mg (91% purity, 0.30 mmol)	10 mg	5 h	2 mL

Entry	molar ratio H_2O_2/oleic acid	temperature (°C)	c (% of epoxide 9, GC/MS)
1	2.2	30	38
2	2.2	50	85
3	1.8	30	42
4	1.8	50	84

Table S2. Effect of the amount of lipase and solvent on the chemo-enzymatic epoxidation of oleic acid

oleic acid	molar ratio H_2O_2/oleic acid	temperature	reaction time
93 mg (91% purity, 0.30 mmol)	1.8	50 °C	5 h

Entry	lipase (mg)	acetonitrile (mL)	c (% of epoxide 9, GC/MS)
5	30	2	96
6	30	6	86
7	10	6	72
8	10	2	98

Conversion of 8-(3-octyloxiran-2-yl)octanoic acid (9) into *threo*-9,10-dihydroxystearic acid (10)

Table S3. Effect of the amount of aqueous 2M sulfuric acid on the ring-opening of epoxystearic acid **9**

epoxystearic acid	acetonitrile/water 3/1	temperature	reaction time
60 mg (0.20 mmol)	4 mL	25 °C	3 h

Entry	molar ratio H_2SO_4/epoxide	c (% of diol 10, GC/MS)
1	0.250	92
2	0.125	69
3	0.065	33

Characterization of 8-((2*SR*,3*RS*)-3-octyloxiran-2-yl)octanoic acid (9)

A suspension of Novozyme 435 (240 mg) in acetonitrile (48 mL), containing oleic acid (2.2 g, 91% purity, 7.1 mmol) and H_2O_2 35 % w/w (1.1 mL, 12.8 mmol), was shaken in an orbital shaker (160 rpm, 50 °C) for 5 h. The enzyme was removed by filtration, and a saturated solution of $NaHSO_3$ (2 mL) was added to the filtrate. Acetonitrile was partially removed by *in vacuo* distillation and then extraction was performed using EtOAc. The organic phase was dried (Na_2SO_4), and concentrated under reduced pressure, to give a solid residue that, after trituration with a hexane, afforded epoxide **9** (1.76 g, 83%). ^{1}H NMR ($CDCl_3$, 400 MHz)[1]: δ = 2.95 – 2.75 (2H, m, *CH*-O-*CH*), 2.35 (2H, t with J = 7.5 Hz, CH_2COOH), 1.70 - 1.15 (26H, m, 13 CH_2), 0.92 - 0.89 (3H, m, CH_3). ^{13}C NMR ($CDCl_3$, 100.6 MHz)[1]: δ = 179.6, 57.5, 57.4, 34.2, 32.0, 29.7, 29.6, 29.4, 29.33, 29.28, 29.1, 27.91, 27.87, 26.70, 26.66, 24.8, 22.8, 14.2. GC/MS (EI) as a methyl ester, obtained by treatment with MeOH and trimethylsilyldiazomethane 10% in hexane, t_r = 22.50 min: m/z (%) = 312 (M+, 1), 199 (11), 171 (15), 155 (100).

Recovery and re-use of Novozyme 435

A suspension of Novozyme 435 (120 mg) in acetonitrile (24 mL), containing oleic acid (1.1 g, 91% purity, 3.5 mmol) and H_2O_2 35 % w/w (0.55 mL, 6.4 mmol), was shaken in an orbital shaker (160 rpm, 50 °C) for 5 h. A saturated solution of $NaHSO_3$ (1 mL) was added, and the enzyme was recovered by filtration, washed first with water and then with acetonitrile. A sample of the filtrate was concentrated, treated with water, extracted with ethyl acetate and derivatized with MeOH and trimethylsilyldiazomethane 10% in hexane for GC/MS analysis. The lipase was stored at 4°C for 18 h, and then the reaction was repeated. Here are the results of four subsequent runs, expressed as percentage of the epoxide present in the reaction mixture as determined by GC/MS analysis: i) 97%, ii) 92%; iii) 87%; iv) 78%.

Oxidative cleavage of 9,10-dioxostearic acid (11) to give a mixture of 9-(nonanoyloxy)-9-oxononanoic acid (12), azelaic (2) and pelargonic acid (3)

A mixture of dioxo derivative **11** (50.0 mg, 0.160 mmol) and H_2O_2 35 % w/w (22 μL, 0.256 mmol) in toluene (2 mL) was stirred at 30°C for 3 h. A saturated solution of $NaHSO_3$ (200 μL) was added, and the reaction mixture was then extracted with ethyl acetate. The organic phase was dried and concentrated under reduced pressure to give a crude mixture containing 51 % of anhydride **12**, besides pelargonic (**3**) and azelaic acid (**2**). The following signals, appearing in the ^{1}H and ^{13}C NMR spectra of the final mixture, could be unambiguously assigned only to compound **12** without

overlapping with other peaks: ^{1}H NMR ($CDCl_3$, 400 MHz) 2.44 (4H, t with J = 7.5 Hz, CH_2COOCOCH_2). ^{13}C NMR ($CDCl_3$, 100.6 MHz): 169.73 and 169.66, 35.4 and 35.3.

Column chromatography separation of azelaic (2) and pelargonic acid (3)

A mixture of crude dioxo derivative **11** (1.50 g, 75% purity, estimated content of compound **12** 3.61 mmol) and H_2O_2 35 % w/w (497 μL, 5.78 mmol) in toluene (30 mL) was stirred at 30°C for 3 h. A saturated solution of $NaHSO_3$ (750 μL) was added, followed by the addition of H_2SO_4 2 M till pH = 2. The reaction mixture was then extracted with ethyl acetate. The organic phase was dried and concentrated under reduced pressure to give a crude mixture containing 95 % (GC/MS) of acids **2** and **3**, which was submitted to column chromatography, eluting with hexane and an increasing quantity of ethyl acetate, to afford pelargonic acid (**3**) (479 mg, 84%) and azelaic acid (**2**) (550 mg, 81%) as pure compounds. The spectroscopic and analytical data of the two compounds were in agreement with those described in the text.

References

[1] Zhao, Y.; Chen, Y.; Newhouse, T. R. Allyl-Palladium-Catalyzed α,β-Dehydrogenation of Carboxylic Acids via Enediolates. *Angew. Chem. Int. Ed.* **2017**, *56*, 12791-13149.
